# Maser: one-stop platform for NGS big data from analysis to visualization

**DOI:** 10.1093/database/bay027

**Published:** 2018-04-13

**Authors:** Sonoko Kinjo, Norikazu Monma, Sadahiko Misu, Norikazu Kitamura, Junichi Imoto, Kazutoshi Yoshitake, Takashi Gojobori, Kazuho Ikeo

**Affiliations:** 1 Center for Information Biology, National Institute of Genetics, 1111 Yata, Mishima, Shizuoka 411-8540, Japan; 2 Hitachi, Ltd, Tokyo, Japan; 3 BITS Co., Ltd, Tokyo, Japan; 4 Graduate School of Agricultural and Life Sciences, The University of Tokyo, Tokyo, Japan; 5 Computational Bioscience Research Center, King Abdullah University of Science and Technology, Thuwal, Saudi Arabia; 6 Department of Genetics, SOKENDAI, Mishima, Japan

## Abstract

A major challenge in analyzing the data from high-throughput next-generation sequencing (NGS) is how to handle the huge amounts of data and variety of NGS tools and visualize the resultant outputs. To address these issues, we developed a cloud-based data analysis platform, Maser (Management and Analysis System for Enormous Reads), and an original genome browser, Genome Explorer (GE). Maser enables users to manage up to 2 terabytes of data to conduct analyses with easy graphical user interface operations and offers analysis pipelines in which several individual tools are combined as a single pipeline for very common and standard analyses. GE automatically visualizes genome assembly and mapping results output from Maser pipelines, without requiring additional data upload. With this function, the Maser pipelines can graphically display the results output from all the embedded tools and mapping results in a web browser. Therefore Maser realized a more user-friendly analysis platform especially for beginners by improving graphical display and providing the selected standard pipelines that work with built-in genome browser. In addition, all the analyses executed on Maser are recorded in the analysis history, helping users to trace and repeat the analyses. The entire process of analysis and its histories can be shared with collaborators or opened to the public. In conclusion, our system is useful for managing, analyzing, and visualizing NGS data and achieves traceability, reproducibility, and transparency of NGS analysis.

**Database URL**: http://cell-innovation.nig.ac.jp/maser/

## Introduction

Thanks to the technological advancements of next-generation sequencing (NGS) and lower pricing (1), the number of NGS data is exponentially increasing (Sequence Read Archive database growth at https://trace.ncbi.nlm.nih.gov/Traces/sra/). Along with this, a variety of analytical tools have been developed for various types of sequencers and experiments. These phenomena generate problems in handling and analyzing big NGS data: (i), to store and manage big data, a large hard disk is required; (ii), analyzing big data requires high levels of computational power; (iii), selecting tools suitable for experiments among a variety of tools is challenging; (iv), it is also difficult for many biologists to handle those tools because most NGS tools are command-line programs. Mastering command-line UNIX/LINUX OS and script languages (e.g. Perl, Python, R, Ruby, shell scripts) entails high learning costs. The difficulty and complexity of NGS data analysis not only increase the burden of analysis but also reduce the traceability, reproducibility, and transparency of the analysis (2,3).

To overcome these issues, we have developed a cloud-based data analysis platform, Maser (Management and Analysis System for Enormous Reads). On Maser, a user can store up to 2 terabytes of data to perform analysis on the Maser server, thus they do not need to prepare a high-spec computer. To make analysis simpler and easier, Maser offers analysis pipelines in which several individual tools are combined as a single pipeline for very common and standard analyses. These pipelines can be run with graphical user interface (GUI) operations, achieving simplified NGS analysis. Maser records all the files used and all the analyses executed, enabling users to trace and repeat the analyses. This leads to analysis traceability and reproducibility. The entire process of analysis and its histories can be shared with collaborators or fully opened to the public, allowing users to clearly demonstrate what analyses were done. This increases the transparency of the analysis.

To further facilitate NGS data analysis, we also developed an original genome browser, Genome Explorer (GE), which visualizes genome assembly and mapping results output from Maser pipelines. The Maser analysis pipelines for mapping work in conjunction with GE, and when executed, these automatically upload the genome and mapping results to the GE that can be viewed on the web. In addition to the mapping results with GE, the visualized results output from the other embedded tools in a pipeline are also collected into a html file, enabling users to see the all visualized results on the web. Therefore, our system is useful for analyzing, viewing, and sharing NGS data and hence reduces some of the burden of NGS data analysis for researchers.

In this report, we introduce the basic functions of the Maser platform and GE that facilitate NGS data analysis and contribute to research in life science.

## Materials and methods

### Registration to Maser and user guide

Maser is publicly available on the Internet with free registration at the User Guide pages for Maser: http://cell-innovation.nig.ac.jp/maser/. After registration, the Maser front page will be accessible: https://cell-innovation.nig.ac.jp/members/maser3. We recommend Google Chrome for use of Maser in both Windows and Macintosh OS.

### Maser data types

The following types of data can be uploaded and used on Maser: sequence data (Fastq, Fasta, sff), mapping results (BAM, sam, maf), gene features (gtf, gff, bed), expression tables (tsv, txt), tree formats (newick) and others. For a full list of all available data types, see the page for ‘Data types’ in the User Guide: http://cell-innovation.nig.ac.jp/maser/ UserGuide/data_types_top_en.html.

### Preset reference genome

Over 160 reference genomes are preset on Maser, including human (hg15-19, 37,38), mouse (mm5-10), rat (rn2-5), chicken (galGal2-4), plants, fungi and bacteria. For a full list of the preset genomes, see the page for ‘Supported genome version and species’ in the User Guide: http://cell-innovation/maser/DataInfo/GEM0000002_en.html.

### Analysis pipelines and run time

There are ∼400 analysis pipelines integrated on Maser. A list of all analysis pipelines, including descriptions and approximate execution times, can be found on the page for ‘All pipelines’ in the User Guide: http://cell-innovation.nig.ac.jp/maser_cgi/cip-pl_list_violin_en.cgi.

### Data release to the public

The projects and/or analysis histories created by users can be opened to the public for the publication of scientific papers. Please contact cip-contact@cello.lab.nig.ac.jp for further details.

## Concept of Maser

The original concept of the development of Maser is to bridge a gap between informaticians (expert of informatics such as tool developers or system engineers) and non-informaticians (who are less familiar with informatics such as general biologists or medical researchers). One of the remarkable differences between them is preference for flexible user interface such as character user interface (CUI; use of command line, LINUX OS and computer languages, such as Shell, Perl, R, Python, Ruby and so on). CUI is compatible with batch execution and is suitable for tool development and implementation, but require high learning cost. On the other hand, non-informaticians usually prefer the GUI operations for intuitive use. To facilitate works for both, Maser is equipped with a pipeline registration system. This is for registering CUI-based tools (alone or in combination) as analysis pipelines, which can be executed by GUI operations. With this system, the burden of tool registration on informaticians is reduced, making it easier to create an analysis pipeline suitable for each type of experiments or change according to user’s opinion. This original concept has led to the current provision of a variety of analysis pipelines according to diverse needs, and furthermore, led to the project-based analysis service offering since 2014. We are aiming to improve our platform and continue the project in the future.

### Maser system configuration and features

**Figure 1.**
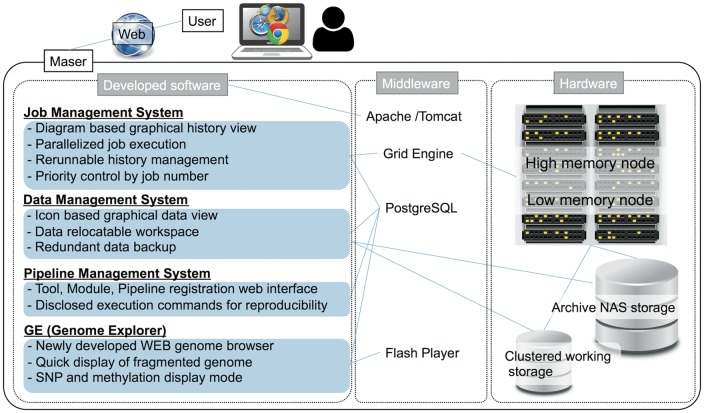
System configuration of Maser.

Maser is designed as a set of four software systems that function at task execution (i) job management, (ii) data management, (iii) pipeline management, (iv) GE (Figure 1). These are implemented in Java and are running on the Apache Tomcat web server. (i) In job management system, Sun Grid Engine is used to job distribution for parallel processing. (ii) The data management system works for creation of a project room (workspace) for storing data, execution of pipelines, icon-based graphical data view, and data backup. (iii) The pipeline management system is designed to control the web interface for registration of individual or combined tools as a pipeline and to disclose all execution commands for reproducibility. (iv) GE is a web-based genome browser. It was developed independent of Maser, but by linking with Maser, it enabled automatic visualization of the mapping result. By rearranging the mapping results in the order of the genome positions and storing the data in the PostgreSQL, GE can quickly display the results of analysis even the genome with poor assembly quality, such as consisting of tens of thousands of contigs/scaffolds. It also supports display of SNP and methylation sites.

Maser hardware consists of a high memory node consisting of 8 CPU core and 144 GB × 10 memory, a low memory node consisting of 8 CPU cores and 72 GB × 10 memory. Maser uses archive NAS storage (slow processing but inexpensive) and clustered working storage (high processing speed but expensive) (Figure 1). In low memory nodes, mapping analysis of sequence reads is performed mainly, and in addition to that, assembling is performed in high memory nodes, by distributed processing. Furthermore, by analyzing with the high-speed working storage eliminates the bottleneck of the disk IO in the parallel computation by load distribution, and by transferring the data to the archive storage two weeks after the analysis were completed realizes cost-effective data analysis and management.

Maser has the following four useful features to be noted.

#### Simple

The first feature is the analysis pipeline. On Maser, several individual tools are combined as a single pipeline for the standard analysis to enable users to conduct a number of analyses with fewer actions. Pipelines are categorized according to experiment design (RNA-seq, ChIP-seq, Bisulfate-seq (BS-seq), Resequencing (Exome-seq), *De novo* genome sequencing, Metagenome, CAGE-seq and SAGE-seq) to make pipeline selection easier.

#### Reproducible

The second feature is the ‘Reanalysis’ button assigned to each analysis. With this button, a user can re-execute the same analysis using the same pipeline and tool option. This function is useful when the user wishes to re-analyze data by partially changing the option settings.

#### Traceable

The third feature is an assignment of unique IDs to each of the files and analysis conducted (‘Request ID’ described in the following section). Maser assigns unique IDs to uploaded files and to output/intermediate files generated by the analysis. The IDs cannot be modified once assigned by Maser, while a file name can be modified as many times as needed. When an analysis pipeline is executed, all file IDs are recorded and tied to a Request ID, enabling users to trace the analysis they have performed. In addition, Request ID can be used as a proof of analysis that defines which files were used and what pipeline was executed with what options. Therefore, the Maser ID system is highly useful to clearly demonstrate the contents of analysis in a scientific paper.

#### Shareable

The fourth feature is the ‘Share’ button that shares the Project Rooms, including the data and analysis histories, with collaborators. The user (the owner of the data) decides whether to share their own data by using the ‘Share’ button. This function facilitates group work and information sharing among collaborative researchers. Users can also release the project or analysis histories to the public with the publication of a scientific paper (see ‘Materials and methods’ section for the detail). This function of Maser brings transparency to the analysis.

## Basic usage of Maser

**Figure 2.**
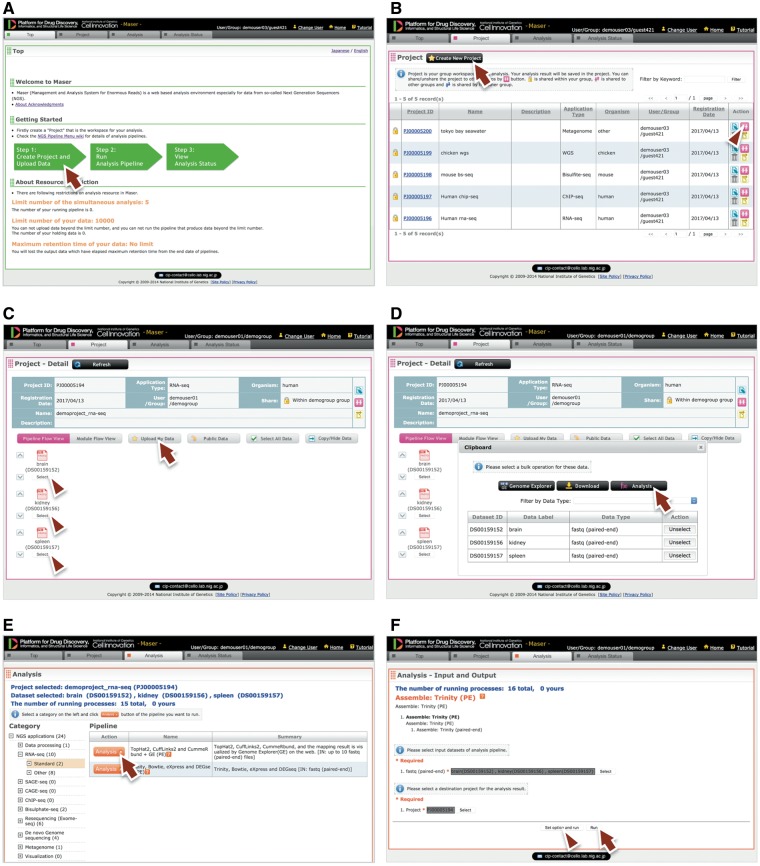

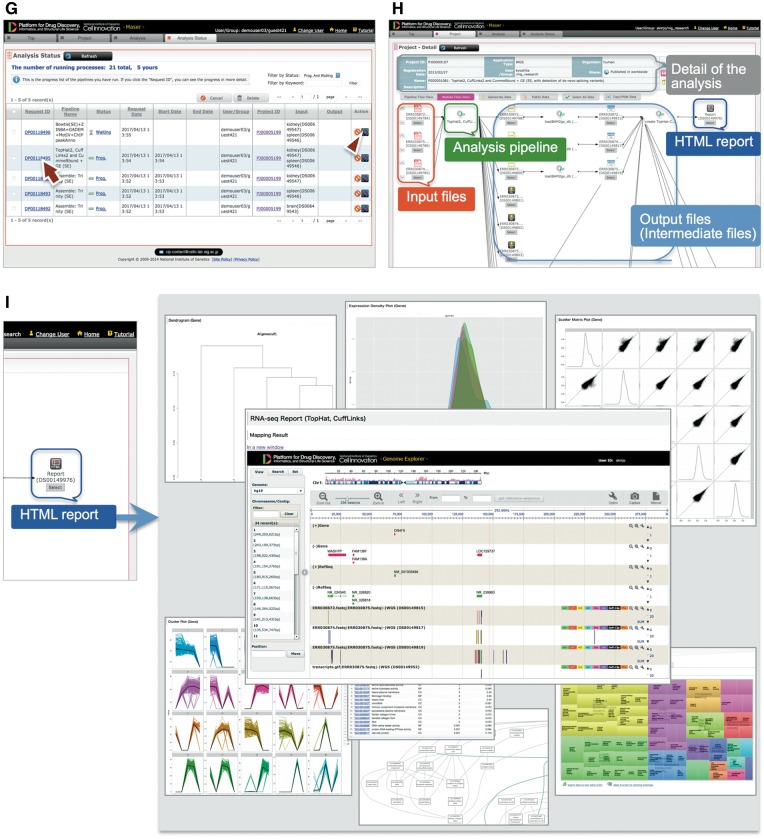
Maser web interface. **(A)** Maser front page. Three green block arrows indicate the main steps to start the analysis. Step 1: Create project and upload data (red arrow), Step 2: Run analysis pipeline, Step 3: View analysis status. **(B)** Project page. Arrow indicates button to create new project and arrowhead indicates icon to share the project with collaborators. **(C)** Project Room view. Upload data files (arrow) and select files used for the analysis (arrowheads). **(D)** By clicking “Analysis” in the new window (arrow), the Maser screen automatically moves onto a list of analysis pipeline **(E)**. **(F)** Option setting screen. There are two buttons, ‘Run’ to start the analysis (arrow) and “Set option and run” (arrowhead) to change the option setting. **(G)** Analysis status page. The Request ID (arrow) is assigned to each analysis. Arrowhead indicates ‘Reanalysis’ icon to repeat the analysis. Clicking the Request ID shows details of the analysis **(H)**. **(I)** A representative example of RNA-seq pipeline analysis results, ‘TopHat2, CuffLinks2 and CummeRbund + GE’. This pipeline produces an html report that contains output files from all the embedded tools (e.g. TopHat, Cufflinks, CummeRbund) and visualized mapping results on GE (see Figure 3).

Performing analysis on Maser is done in three steps, as shown in green block arrows on the front page (Figure 2A). Step 1: Create Project Room (workspace) to upload data, Step 2: Run analysis pipeline, and Step 3: View analysis status to check the progress of analysis.

Step 1: To get started, a user has to create a ‘Project Room’ for uploading data and performing analysis. First, move to the Project tab from the front page by clicking the left-most green block arrow (Figure 2A, arrow) and create a Project Room using the ‘Create new project’ button (Figure 2B, arrow). Next, open the newly created Project Room and upload data using the ‘Upload Data’ button (Figure 2C, arrow). To upload data, the file type (e.g. Paired-end Fastq, Single-end Fastq, Fasta or BAM format) should be specified. See the ‘Materials and methods’ section for detail on the file types available on Maser.

Step 2: To start the analysis, choose input files by clicking the ‘Select’ button (Figure 2C, arrowhead), then click the ‘Analysis’ button that appears in the new window (Figure 2D, arrow). Now the Maser screen moves onto the ‘Analysis’ list (Figure 2E) and only the executable pipelines (according to the file format of the input data) are shown in the pipeline list. Choose the analysis pipeline (Figure 2E, arrow), then click ‘Run’ to start the analysis (Figure 2F, arrow). Alternatively, a user can change options from the ‘Set option and run’ button if necessary (Figure 2F, arrowhead).

Step 3: When the analysis is running, the Maser screen automatically moves onto the ‘Analysis status’ page and the user can see the execution status of the analysis: Waiting, Progress, End, Abort or Cancel (Figure 2G). Clicking Request ID assigned to each analysis (Figure 2G, arrow) shows details of the analysis history (Figure 2H). In this view, input file(s), analysis pipeline and resultant output file(s) are connected by arrows, enabling users to confirm the relationships between input/output file(s) and the analysis conducted.

As a result, the Maser pipeline produces a report that summarizes the output of all the embedded tools (Figure 2I). For example, on the RNA-seq pipeline, ‘TopHat2, CuffLinks2 and CummeRbund + GE’ creates a report that contains the results of mapping by TopHat2, gene prediction and differential gene expression analysis by CuffLinks2, and visualization of CuffLinks2 outputs by cummeRbund. Furthermore, the result of mapping by TopHat2 is displayed in a web browser using GE (see below).

### GE: visualization of the assembly and mapping results

**Figure 3.**
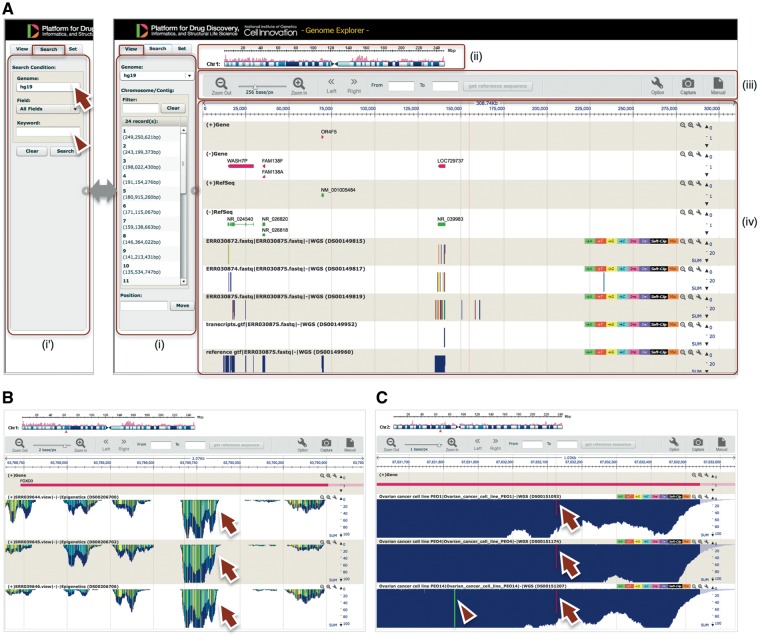
GE implemented on Maser. **(A)** GE display is composed of four components: (i) Genome box, (ii) Chromosome bar, (iii) Control menu bar and (iv) Mapping view. (i) The left Genome box shows a list of chromosomes or scaffolds of reference genomes the user has selected. The user can specify the chromosome and position to be displayed or search a specific gene by keywords (Entrez gene ID, Refseq ID, gene name and symbol) in the Search tab (i'). (ii) The top Chromosome bar shows the location in the chromosome displayed. (iii) The second top Control menu bar has multiple functions. There are buttons to zoom in and out of the chromosome, shift left or right on the chromosome region and retrieve any sequences in the range specified in the ‘From’ and ‘To’ boxes. The ‘Option’ button is used for selecting datasets displayed on the mapping view and changing plot format. (iv) The Mapping view is composed of multiple tracks that show the position of Entrez gene, Refseq transcripts, and mapping results. **(B)** Magnified mapping view with the detected methylation sites output from the BS-seq pipeline. The methylation patterns of all CpG, CHH and CHG regions throughout the genome are shown in cyan, green, and yellow, respectively (arrows). **(C)** Magnified mapping view with the detected SNVs and indels output from the Resequencing (Exome-seq) pipeline. The arrows and arrowhead indicate T variant (red vertical bar) and A variant (green vertical bar), respectively.

GE is a web-based genome browser that we originally developed to quickly visualize the results of genome assembly and mapping within Maser (Figures 2I and 3). The Maser analysis pipelines automatically produce visualized mapping results on GE, as seen in Figure 2I. More than 50 species of reference genomes including human, mouse, rat and other model organisms are preset on GE and the user can simply choose one of them from a drop-down list (Figure 3A, arrow).

In addition to the visualizing function, GE is also used as a simple database for genome assembly and gene annotation. A user can search a specific gene with keywords (Figure 3A, arrowhead), such as Entrez gene ID, Refseq ID, gene name and symbol, to be displayed on GE. A user can also embed arbitrary keywords in GE upon registering newly assembled custom genome and annotation information on GE. Therefore this function of GE is useful to publicly release *de novo* genome assembly and annotation.

### Standard pipelines

Table 1 lists the representative pipelines implemented on Maser. Pipelines are divided into the following 10 categories according to analysis steps or experimental types, and further subdivided into standard pipelines (recommended, basic or full-course pipeline for primary analysis) and others (individual tools for additional analysis). Among ∼400 pipelines implemented on Maser, we carefully selected pipelines to be offered as standard pipelines based on use frequency in Maser and scientific papers. The embedded tools in the pipelines are uploaded as necessary when new versions are released. The details of these standard pipelines are described below. In addition, the analysis workflow can be seen on the website ‘NGS Pipeline Menu’ at http://cell-innovation.nig.ac.jp/maser/.

**Table 1.** Standard pipelines implemented on Maser

Category	Pipeline name	Current version		RRID
1. Data processing	FastQC	v0.11.5		SCR_014583
	Flexbar	v2.4		SCR_013001
2. RNA-seq	TopHat2, CuffLinks2 and CummeRbund + GE	TopHat	v2.0.6	SCR_013035
		CuffLinks	v2.0.2	SCR_014597
		Cuffmerge	v2.0.2	SCR_015688
		Cuffdiff	v2.0.2	SCR_001647
		CummeRbund	v2.0	SCR_014568
	Trinity, Bowtie, eXpress and DEGseq	Trinity	r2012-10-05	SCR_013048
		Bowtie	v0.12.8	SCR_005476
		eXpress	v1.2.2	SCR_006873
		DEGseq	v1.2.2	SCR_008480
3. SAGE-seq	TopHat2, CuffLinks2 and CummeRbund + GE for SAGE	TopHat	v2.0.6	SCR_013035
		CuffLinks	v2.0.2	SCR_014597
		Cuffmerge	v2.0.2	SCR_015688
		Cuffdiff	v2.0.2	SCR_001647
		CummeRbund	v2.0	SCR_014568
4. CAGE-seq	nAnT-iCAGE (RIKEN CLST)	Fastx toolkit	v0.0.14	SCR_005534
		TopHat	v2.0.14	SCR_013035
	RECLU	EdgeR	v2.6.7	SCR_012802
5. ChIP-seq	Bowtie-MACS2-GADEM-MotIV-ChIPeakAnno	Bowtie	v1.1.2	SCR_005476
		MACS2	v2.0.10	SCR_013291
6. Bisulphate-seq	Bismark base multi sample comparison pipeline	Bismark	v0.14.5	SCR_005604
	BMap base multi sample comparison pipeline	Bmap	v1.1	SCR_016044
7. Resequencing (Exome-seq)	BWA, GATK and snpEff + GE	BWA	v0.7.5	SCR_010910
		GATK	v2.1.13	SCR_001876
8. *De novo* Genome sequencing	SOAPdenovo2	SOAPdenovo	v2.04	SCR_014986
	SOAPdenovo2 scaf and GapCloser	SOAPdenovo	v2.04	SCR_014986
		GapCloser	v.1.12	SCR_015026
	Annotation after assembling	Augustus	v2.7	SCR_008417
		BLAST	v2.2.26+	SCR_008419
9. Metagenome	SOAPdenovo2	SOAPdenovo	v2.04	SCR_014986
	mapped read count with BWA-mem	BWA	v0.7.4	SCR_010910
	blastn for silva, NT or CAMERA database	BLAST	v2.2.26+	SCR_008419
				SCR_006423
10. Visualization	loadBAM2ge_db	v1		SCR_015951
	loadBED2ge_db	v1		SCR_015996
	loadGffToGe_db	v1		SCR_015997
	loadGtfToGe_db	v1		SCR_015998
	regist custom genome	v1		SCR_015999

1. Data processing: This category contains pipelines for quality checking and data cleaning of Fastq files before starting the analysis. FastQC (4) is used for quality checking and Flexbar (5) for data cleaning, such as trimming and filtering out adapter sequences and low-quality reads.
2. The RNA-seq category contains two standard pipelines: one begins with mapping NGS reads to the reference genome with TopHat2 (6) and the others begin with assembling the reads with Trinity (7,8) for the species with no reference genome. The former pipeline, ‘TopHat2, CuffLinks2 and CummeRbund + GE’, subsequently predicts transcripts, estimates the abundance of each transcript to test differentially expressed genes (DEGs), and visualizes the resultant outputs with the Cufflinks suite (9). The latter pipeline, ‘Trinity, Bowtie, eXpress and DEGseq’, subsequently maps raw NGS reads to transcriptome assembly using Bowtie (10) to estimate the abundance of transcriptome using eXpress (11) and test DEGs with DEGseq (12). In both pipelines, the resultant DEGs are subjected to a gene ontology (GO) enrichment test using GOseq (13) and visualization by REVIGO (14). These pipelines finally produce an html report that summarizes the output of all the embedded tools (Figure 2I).
3. The SAGE-seq category has a pipeline that is developed by reference to the RNA-seq pipeline. This pipeline starts from mapping the reads to a reference genome using TopHat2 and estimates the abundance of transcripts using Cufflinks. The difference from the RNA-seq pipeline is that the Cufflinks option to normalize read counts based on gene length is turned off because the SAGE-seq library generates only a single fragment for a transcript (3’-most fragment of the cleaved cDNA with a restriction enzyme) in principle (15). The subsequent analysis statistically detects DEGs and performs k-means clustering and the GO enrichment test, the same as the RNA-seq pipeline.
4. CAGE-seq pipelines are developed by the RIKEN Institute (16) and implemented into Maser. Briefly, the ‘nAnT-iCAGE’ (17) pipeline processes CAGE reads and maps those reads to a reference genome using TopHat. Next, using the mapping results, the pipeline ‘RECLU’ (18) estimates transcription start sites (TSSs) based on the cluster of mapped CAGE tags (equivalent to gene structure prediction) and compares the abundance of tags for each TSS between control and case inputs, then detects differentially expressed TSS using edgeR (19). In addition, RECLU searches binding motifs adjacent to the differentially expressed TSS using multiple tools, such as GLAM2 (20), Weeder (21) and DREME (22), to infer transcription factors that might be involved in the differential expression of genes between datasets. The supported reference genome with this pipeline is restricted to the preset genomes, such as human, mouse, and rat.
5. The ChIP-seq category contains a single pipeline that executes the mapping with Bowtie2 (23), peak calling with MACS (24). The pipelines subsequently search binding motifs with GADEM for a *de novo* motif (25) and MotIV for a known motif (26) in the defined peaks, and the nearest neighbor gene to those motifs (annotation).
6. The BS-seq category contains two pipelines that differ in the mapping tool they use: Bismark (27) or BMap (28). Both pipelines map the bisulfate NGS reads to a reference genome and call modified DNA bases. The results of read mapping and the detected methylation sites are displayed on GE (Figure 3B).
7. The Resequencing (Exome-seq) category contains a pipeline that predicts single-nucleotide polymorphisms and indels. This pipeline initially aligns NGS reads to a reference genome with BWA (29) and calls genomic variants with GATK. The called variants, including single-nucleotide variants (SNVs) and short Insertion/Deletion of bases (indels), are annotated by snpEff (30), which predicts the influence level of amino acid changes. The results of read mapping and the detected SNVs and indels are displayed on GE (Figure 3C).
8. *De novo* genome sequencing has three standard pipelines for assembling, scaffolding, and gene annotation (Table 1). The first two pipelines produce genome assembly (contigs and gap-closed scaffold sequences) using SOAPdenovo (31). The last pipeline estimates gene structure with Augustus (32), and performs homology searches of the resultant gene models with BLAST+ blastp (33) against UniProt and the National Center for Biotechnology Information (NCBI) non-redundant protein sequences (nr) database to infer gene function. In addition to SOAPdenovo as a standard pipeline, Maser is also equipped with other major assemblers, such as Ray (34), MIRA (35) and Platanus (36).
9. The metagenome category contains five pipelines for assembling, mapping, and annotation. For assembling, SOAPdenovo is prepared as a standard pipeline, but other assemblers such as those of *de novo* genome sequencing are also available. The second pipeline, ‘mapped read count with BWA-mem’, maps the reads onto assembled contig sequences using BWA-MEM (37) and calculates the abundance of reads in each contig. Pipelines for annotation perform homology searches of the assembled contigs or raw reads with BLAST+ blastn (33) against the SILVA SSU-rRNA database (38), microbe DNA database provided by CAMERA (39), and NCBI non-redundant protein sequences (nt) database. The output files (blastresult with text format) can be used for further analysis, such as taxonomical contents of sample with the MEGAN program (40).
10. The visualization category contains pipelines for uploading mapping results (BAM/BED format) and gene feature files (Gff/Gtf format), and registering custom genomes (Fasta format) to GE (Table 1). Although visualization on GE is automatically performed by standard pipelines as mentioned above, hence those pipelines will not usually be needed, they are available if users wish to create newly assembled custom genomes.

### Comparison of Maser and GE with other platforms and genome browsers

In order to improve understanding on Maser, a comparison table of Maser with Galaxy (2) is shown in Table 2. Basically, both Maser and Galaxy work with GUI and have functions for data management, analysis history record, reanalysis and data sharing. One of the major differences between Maser and Galaxy is the number of workspaces that a user can have. In Maser users can create multiple workspaces (‘project rooms’) to upload data and conduct analyses (as listed in Figure 2B). This is useful and less confusion when conducting analyses with different data in parallel. The other difference is the graphical view of the workspace and analysis record (‘Graphical data/analysis record display’ in Table 2). In Maser, input file(s), analysis pipeline, and resultant output file(s) are displayed by icons and they are connected by arrows and displayed in a workspace (Figure 2C and H), while in Galaxy those files are only listed separately in the analysis history. This Maser graphical display helps users, especially who are unfamiliar with the analysis, to grasp the relationships between input/output file(s) and the analysis conducted.

**Table 2.** Comparison table of Maser with Galaxy

		Maser	Galaxy
Basic			
Web-based GUI		✓	✓
Workspace (Project room)		Multiple	Single
Graphical data/analysis record display		✓	
Data upload			
Local file		✓	✓
Direct link to public database			✓
Data analysis			
Provided pipeline/tool		✓	✓
Pipeline/tool classification		Experiment type	Tool/file/analysis type
User’s custom made pipeline/workflow			✓
Sharing			
With individual user		✓	✓
Open to the public		✓	✓

Regarding the data upload, while Galaxy has function to acquire data file through direct link to public database, Maser does not (‘Direct link to public database’ in Table 2). Although Maser has a function to acquire data from the SRA database, this is not disclosed to general users to prevent oppression of disc capacity. Therefore, as for the public data, we ask users to download them to local computer then upload to the Maser server. Similarly, Maser also has a system to create custom-made analysis pipeline with graphical interface, but to limit the increase of pipelines, we do not allow general users this function. On the other hand, in Galaxy, users can create custom workflows freely (‘User’s custom made pipeline/workflow’ in Table 2). Instead of allowing free registration of pipeline/workflow by users, Maser offers the carefully selected standard pipelines that combine the NGS tools commonly used for each experiment type (‘Pipeline/tool classification’ in Table 2), helping pipeline selection by users who are unfamiliar with NGS tools and analysis.

Table 3 shows a comparison table of GE with other genome browsers, Trackster (41), UCSC genome browser (42) and IGV (43) that work with Galaxy. In all genome browsers, mapping result (e.g. BAM/SAM file) are automatically uploaded and displayed on the web (GE, Trackster, UCSC) or local application (IGV). In addition to that, GE has functions of gene name search, DNA sequence acquisition, and a color-coded display for SNP and Bisulfite site. Therefore, from the viewpoint of visualization of whole genome, GE has combined functions of the external browsers (Table 3). On the other hand, Galaxy’s Trackster is more adapt to display selective gene/region rather than whole genomes to work quickly in cooperation with analysis tools (e.g. parameters of analysis tools can be changed while displaying with viewer in real time) (41). This interactive visualization with data analysis is useful for more complex and detailed analyzes.

**Table 3.** Comparison table of GE with other genome browsers

	GE	Trackster	UCSC	IGV
Connection with Maser or Galaxy				
Availability on Maser (M) or Galaxy (G)	M (built-in)	G (built-in)	G (external)	G (external)
Automatic loading of mapping result	✓	✓	✓^a^	✓^a^
Basic				
Installation not required	✓	✓	✓	
Gene name search	✓		✓	✓
DNA sequence acquisition	✓		✓	✓
Display for individual analyses				
SNP color-coded display	✓		✓	✓
Bisulfate color-coded display	✓			✓
Interactive visualization with data analysis		✓		

a When used with Galaxy.

In conclusion, it could say that Maser realized a more user-friendly analysis platform for beginners by improving graphical display and providing the selected standard pipelines that work with built-in genome browser. While, Galaxy is suitable for users who are more familiar with NGS analysis to perform highly flexible analyzes.

### Usage status of Maser and its application to medical research

Maser was released in 2010 with the aim of supporting analysis of massive NGS data, and currently has ∼700 registered users. Some of those users have achieved solid results (44–48). In addition, we began offering analysis of medical research services in 2014 using Maser under the Platform Project for Supporting Drug Discovery and Life Science Research from the Japan Agency for Medical Research and Development. We have been involved in 177 medical research projects using RNA-seq, ChIP-seq, Exome-seq and other applications, yielding valuable results (49–51).

## Conclusion and future directions

We have developed Maser to help analyze the huge amounts of NGS data. Maser provides a place (Project Room) to keep up to 2 terabytes of data for each user and conducts analysis using a number of NGS tools on the Maser server. For ease of analysis, Maser offers standard pipelines and a newly developed genome browser (GE) that automatically visualizes the results of assembly and mapping. These features could help users both with and without bioinformatics skills to analyze NGS data and to make new discoveries in their research. As a future direction, we are developing analysis pipelines required for the study of comparative genomics and population genetics responding to increased demands. To quickly respond to users’ requests, we designed a system for creating a custom-made pipeline. We hope that our system assists researchers and facilitates basic research in life science.

## Funding

This work was partially supported by the Ministry of Education, Culture, Sports, Science and Technology, Japan (MEXT) grant [code 09002013] and by Agency for Medical Research and Development (AMED) grant [JP16am0101058j0003] and [JP17am0101001].
